# Cancer stemness and progression: mitochondria on the stage

**DOI:** 10.18632/oncotarget.6244

**Published:** 2015-10-26

**Authors:** Paola Chiarugi, Persio Dello Sbarba

**Affiliations:** Department of Experimental and Clinical Biomedical Sciences, Università degli Studi di Firenze, Florence, Italy

**Keywords:** cancer stemness, OXPHOS, cancer metabolism

The idea that mitochondrial functions correlate with tumor malignancy recently arose together with the indication that respiration and processing of different nutrients, as well as the generation of reactive oxygen species, play a mandatory role during key steps of tumor progression. While the Warburg scenario, depending on the availability of glycolytic intermediates capable to fuel anabolic pathways, has been associated with the metabolic demand of growing cells, a metabolic reprogramming to enhanced mitochondrial function has been related to increased aggressiveness. Indeed, mitochondrial oxidants are mandatory to engage epithelial/mesenchymal transition [1], while oxidative phosphorylation and ability to exploit nutrients by respiration sustain dormancy, resistance to therapy and tumor relapse [2].

In line with the above idea, in three very recent OncoTarget papers of Michael Lisanti's laboratory, the mitochondria-addicted cancer phenotype has been correlated with the achievement of stem-like traits [3-5]. They found that the number of mitochondria, as revealed by MitoTracker staining, correlates with stemness, suggesting that cancer stem cells (CSC) may be selected via mitochondrial staining. Noteworthy, a relatively large size of cancer cells was also found associated with stemness and mitochondrial number/function [3]. The latter observation is in contradiction with the idea that biomass increase correlates with the Warburg metabolic profile, which supports anabolic pathways in terms of aminoacid and nucleotide synthesis. Such a paradox is only apparent, as pyruvate kinase M2 (PKM2), a key regulator of the accumulation of glycolytic intermediates needed for cell growth and historically associated with a Warburg profile, is also an upstream regulator of the transcriptional pathway leading to the generation and functional upgrading of mitochondria [1]. Thus, PKM2 lies at the crossroads between biomass accumulation and mitochondria functionalization. On the other hand, Myc has been involved in cell reprogramming towards the mitochondria-based glutamine metabolism, as well as in the control of protein biosynthesis leading to biomass increase. Both observations reveal that the link between biomass increase and mitochondrial functions is strong, although clearly underestimated at present. Lisanti's group strengthened the association of the mitochondria-rich/anabolic/motile phenotype with stem cell traits using a telomerase expression-based model. High telomerase expression was found correlated with number/function of mitochondria and cell size/biomass [4]. Nutrient sensing through sirtuins and PGC1α-mediated increased mitochondrial biogenesis are likely to play a central role in the acquisition of the mitochondria-rich/stem-like/anabolic phenotype, and therefore to support CSC resistance to therapy [6]. Finally, FGF3 and Wnt1 secreted by neighboring cells are able to reprogram cancer cells towards this phenotype, which is therefore apparently under control of microenvironment [5].

The finding that high mitochondrial mass is associated with increased tumor initiation frequency but not with increased tumor (mammosphere) size [3] point to the involvement of mitochondrial function in the maintenance of CSC rather than in their downstream clonal expansion. This impacts on the question of whether the balance of functional phenotypes within the CSC compartment is dynamically regulated by changes of metabolic profile. We found that Chronic Myeloid Leukemia (CML) cells are capable to expand under severe hypoxia as long as (anaerobic) glycolysis and its anabolic effects are sustained by glucose availability. Glucose exhaustion in hypoxia, while suppressing CML cell population expansion, leads to the selection of a cell subset endowed with a completely therapy-resistant CSC potential. The onset of resistance parallels the fact that, while the oncogenetic BCR/Abl protein responsible for CML is expressed until glucose is available, it is suppressed when glucose is exhausted. This was the first study to link metabolism to the loss of oncogene addiction (7 and references therein). Thus, a glycolysis-independent CSC phenotype seems to foster CML resistance to therapy. According to the Lisanti and coworkers’ findings, it is straightforward to hypothesize that this CSC subset of CML rely on enhanced mitochondrial activity and on the oxidation of substrates other than glucose. On the other hand, the relationship of telomerase expression to mitochondrial activity [4] may provide an intriguing explanation to the fact that in hypoxia stem cell potential is maximal in normal hematopoietic cells which have undergone one mitotic cycle, while it is lower in cells which have either remained quiescent or undergone more than one cycle [7 and references therein]. It is tempting to speculate that an increased mitochondrial function paralleles the activation (from quiescence) of stem cells to exploit maximally their potential (highest telomerase activity), which is instead rapidly exhausted during the following glycolysis-driven clonal expansion.

**Figure:**
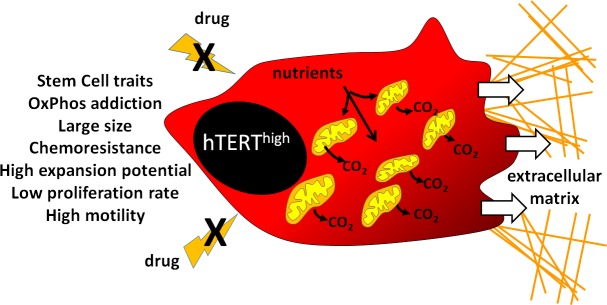


Collectively, the papers recently published by Lisanti's group together with current literature point to the existence, among cancer cell subsets, of a phenotype in which several features (as shown in the Figure) are intimately associated: i) addiction to mitochondrial functions, ii) activation of anabolic pathways, iii) achievement of stem-like traits, iv) resistance to stress and therapy, v) ability to undergo epithelial/mesenchymal transition. Interestingly, this complex phenotype is deeply influenced by microenvironmental factors, including cancer-associated “supporting” cells such as fibroblasts or macrophages (1), cytokines [5] and oxygen/glucose shortage [7]. Needless to say, extensive investigation is still needed to identify common molecular pathways driving the success of the mitochondria-rich/anabolic/motile CSC, a necessary step in order to define a targeted and effective therapeutic strategy against such a strongly malignant tumor cell subset.
